# Supplementary material for: Covalent narlaprevir- and boceprevir-derived hybrid inhibitors of SARS-CoV-2 main protease
Source: Nat Commun. 2022 Apr 27;13:2268. doi: 10.1038/s41467-022-29915-z (PMC9046211; doi:10.1038/s41467-022-29915-z)
Supplement: Supplementary file 1 — Supplementary Information [file 41467_2022_29915_MOESM1_ESM.pdf]

Supplementary Information

Covalent narlaprevir- and boceprevir-derived hybrid inhibitors of SARS-CoV-2 main protease

Daniel W. Kneller,[1] Hui Li,[2] Gwyndalyn Phillips,[1] Kevin L. Weiss,[1] Qiu Zhang,[1] Mark A. Arnould,[2] Colleen B. Jonsson,[3,4,5] Surekha Surendranathan,[5] Jyothi Parvathareddy,[5] Matthew P. Blakeley,[6] Leighton Coates,[7] John M. Louis,[8] Peter V. Bonnesen[2]* and Andrey Kovalevsky[1]*

[1]*Neutron Scattering Division, Oak Ridge National Laboratory, Oak Ridge, TN, 37831, USA*

[2]*Center for Nanophase Materials Sciences, Oak Ridge National Laboratory, Oak Ridge, TN, 37831, USA*

[3]*Department of Microbiology, Immunology and Biochemistry, University of Tennessee Health Science Center, Memphis, TN 38103, USA*

[4]*Institute for the Study of Host-Pathogen Systems, University of Tennessee Health Science Center, Memphis, TN, USA*

[5]*Regional Biocontainment Laboratory, The University of Tennessee Health Science Center, Memphis, TN 38105, USA*

[6]*Large Scale Structures Group, Institut Laue–Langevin, 71 Avenue des Martyrs, 38000 Grenoble, France*

[7]*Second Target Station, Oak Ridge National Laboratory, Oak Ridge, TN, 37831, USA*

[8]*Laboratory of Chemical Physics, National Institute of Diabetes and Digestive and Kidney Diseases, National Institutes of Health, DHHS, Bethesda, MD 20892-0520, USA*

* To whom correspondence should be addressed:
Peter Bonnesen: bonnesenpv@ornl.gov, Andrey Kovalevsky: kovalevskyay@ornl.gov

Supplementary Table 1. Crystallographic data collection and refinement statistics for the joint X-ray/neutron structure of SARS-CoV-2 M^{pro} in complex with BBH-1.

	**M^{pro}/BBH-1** PDB ID 7TDU	
**Data collection:**	**Neutron**	**X-ray**
Beamline/Facility	LADI-DALI (ILL)	Rigaku HighFlux HomeLab
Space group	I2	
Cell dimensions:		
a, *b*, *c* (Å)	55.02, 81.22, 88.83	
α, β, γ (°)	90, 96.7, 90	
Resolution (Å)	44.04 – 2.20 (2.32 – 2.20)	59.75 – 1.85 (1.92 – 1.85)
No. reflections measured	48579 (5302)	171591 (16994)
No. reflections unique	15471 (1840)	32013 (3127)
R_{merge}	0.161 (0.367)	0.076 (0.671)
R_{pim}	0.094 (0.223)	0.037 (0.313)
$CC_{1/2}$	0.986 (0.825)	0.991 (0.598)
$<I / \sigma I>$	7.9 (2.1)	13.6 (1.4)
Completeness (%)	78.7 (64.2)	96.6 (94.2)
Redundancy	3.1 (2.9)	5.4 (5.4)
**Refinement:**	**Joint XN**	
Resolution (neutron, Å)	40 – 2.20	
Resolution (X-ray, Å)	40 – 1.85	
Data rejection criteria	no observation & |F|=0	
Sigma cut-off	2.50	
No. reflections (neutron)	13060	
No. reflections (X-ray)	28704	
R_{work} / R_{free} (neutron)	0.236 / 0.257	
R_{work} / R_{free} (X-ray)	0.196 / 0.210	
R_{work} / R_{free} (joint XN)	0.210 / 0.226	
No. atoms		
Protein, including H and D	4678	
BBH-1	91	
Water	441 (i.e. 147 D_2O molecules)	
B-factors		
Protein	32.4	
BBH-1	28.0	
Water	50.1	
R.M.S. deviations		
Bond lengths (Å)	0.010	
Bond angles (°)	1.16	

Supplementary Table 2. Data reduction and refinement statistics for the room temperature X-ray crystal structures of SARS-CoV-2 M^{pro}-inhibitor complexes used in this study. Values in parentheses are for the highest-resolution shell.

	**M^{pro}/BBH-2** PDB ID 7TEH	**M^{pro}/NBH-2** PDB ID 7TFR	**M^{pro}/PF-07321332** PDB ID 7SI9
**Data collection:**		**X-ray (in-house)**	
Diffractometer		Rigaku HighFlux, Eiger R 4M	
Space group	I2	I2	I2
Wavelength (Å)	1.5406	1.5406	1.5406
Cell dimensions:			
a, *b*, *c* (Å)	55.05, 81.00, 88.75	54.99, 80.97, 88.66	52.54, 81.84, 91.74
α, β, γ (°)	90, 96.8, 90	90, 96.9, 90	90, 95.3, 90
Resolution (Å)	59.6 – 1.80 (1.87 – 1.80)	59.6 – 1.80 (1.87 – 1.80)	60.95 – 2.00 (2.07 – 2.00)
No. reflections unique	33593 (3228)	35735 (3559)	25606 (2516)
R_{merge}	0.037 (0.379)	0.046 (0.479)	0.087 (0.716)
R_{pim}	0.017 (0.210)	0.022 (0.236)	0.041 (0.331)
$CC_{1/2}$	0.998 (0.805)	0.991 (0.817)	0.985 (0.548)
$\langle I / \sigma I \rangle$	33.4 (3.54)	25.92 (2.38)	14.50 (1.31)
Completeness (%)	93.6 (90.0)	99.9 (99.4)	97.7 (95.8)
Redundancy	5.5 (4.3)	5.4 (4.9)	5.6 (5.5)
**Refinement:**			
R_{work} / R_{free}	0.1551 / 0.1856	0.1579 / 0.1831	0.1716 / 0.2067
B-factors			
Protein	37.07	39.12	39.90
Ligand	32.31	38.04	52.42
Water	44.33	44.96	42.00
R.M.S. deviations			
Bond lengths (Å)	0.014	0.017	0.008
Bond angles (°)	1.297	1.448	0.846
All atom clashscore	2.72	1.86	1.88

Supplementary Table 3. Summary of protonation states and corresponding electric charges of the ionizable residues in the SARS-CoV-2 M^{pro} active site observed in four XN structures.

Residue	**M^{pro} ligand-free** (PDB ID 7JUN)		**M^{pro}-Telaprevir** (PDB ID 7LB7)		**M^{pro}-Mcule-5948770040** (PDB ID 7N8C)		**M^{pro}/BBH-1** (PDB ID 7TDU)	
	Charge	Species	Charge	Species	Charge	Species		
$Cys145_{cat}$	–1	Thiolate (-S^{-})	0	S-C-OD (hemithioketal)	0	Thiol (-SD)	0	S-C (hemithioketal)
$His41_{cat}$	+1	Nδ1-D, Nε2-D	0	Nδ1-D	0	Nε2-D	0	Nδ1-D
His163	0	Nδ1-D	+1	Nδ1-D, Nε2-D	+1	Nδ1-D, Nε2-D	+1	Nδ1-D, Nε2-D
His164	+1	Nδ1-D, Nε2-D	0	Nδ1-D	0	Nε2-D	0	Nε2-D
His172	0	Nε2-D	0	Nε2-D	0	Nε2-D	0	Nε2-D
**Net charge**	**+1**		**+1**		**+1**		**+1**	

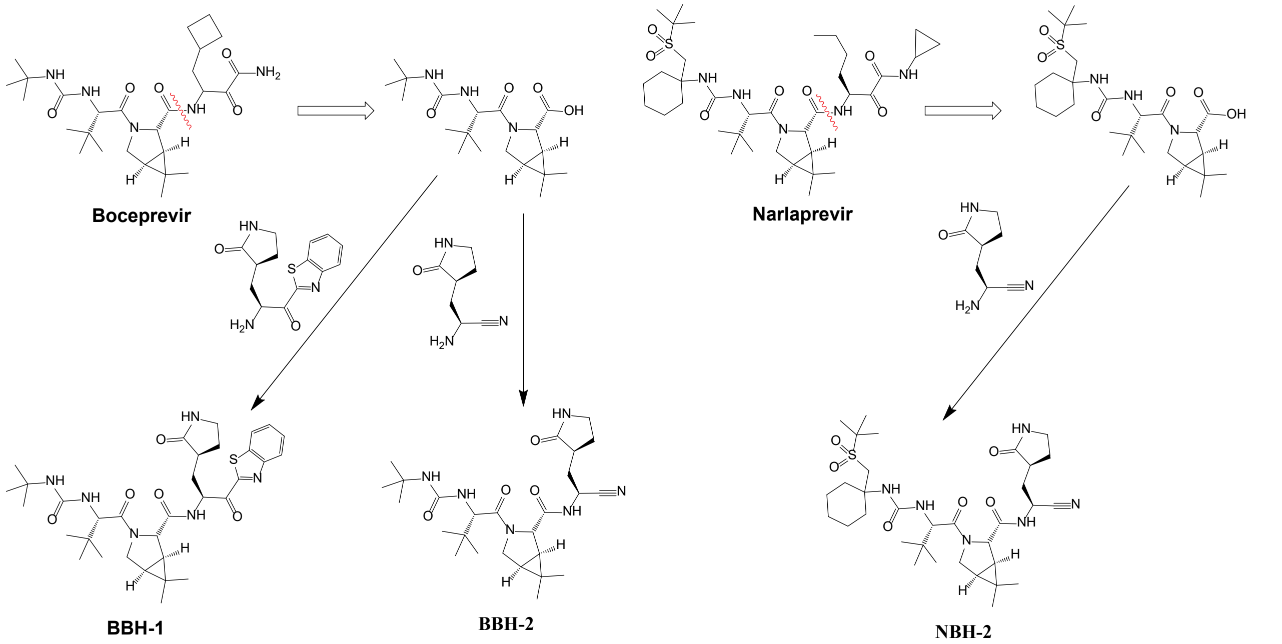

Supplementary Figure 1. Strategy for the syntheses of BBH-1 and BBH-2 from boceprevir fragment, and of NBH-2 from narlaprevir fragment.

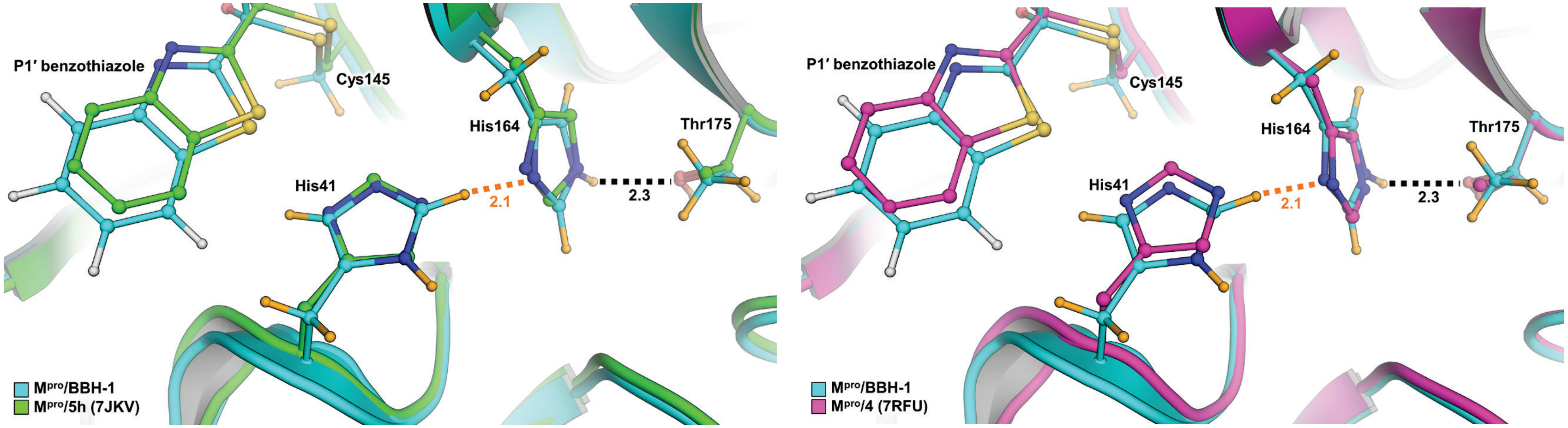

Supplementary Figure 2. Comparison of His41 position between X-ray crystal structures of M^{pro} in complex with inhibitors possessing a P1′ benzothiazole group with XN M^{pro}/BBH-1

Superposition by least-square-fit of Cα atoms. Hydrogen bonds shown as black dotted line. C-D···N interactions are shown as orange dotted line. Distances are in Ångstrom.

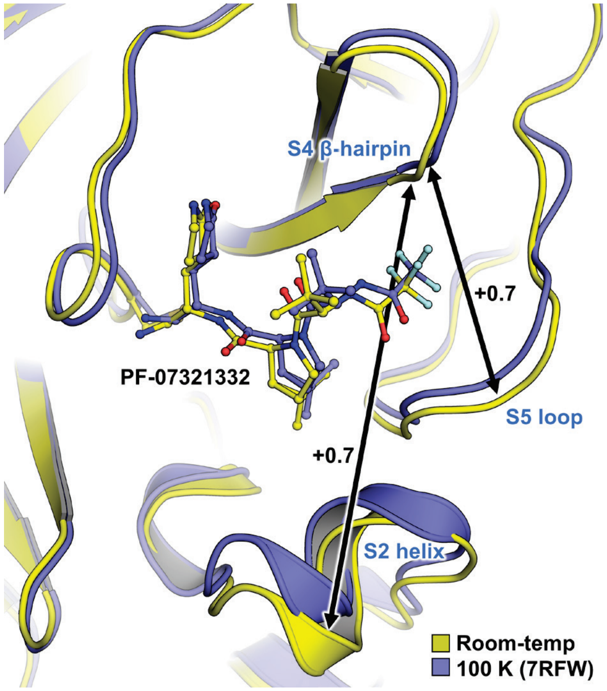

Supplementary Figure 3. Comparison of M^{pro}/PF-07321332 (nirmatrelvir) X-ray crystal structures collected at room and cryogenic temperatures

Superposition by least-square-fit of Cα atoms. Arrows indicate active site expansion observed in the room-temperature structure (yellow) compared to 100 K structure (purple). Distance in angstroms measured between Cα atoms of residues 46 & 168 for S2 helix:S4 β-hairpin span and residues 168 & 190 for the S4 β-hairpin:S5 loop span.

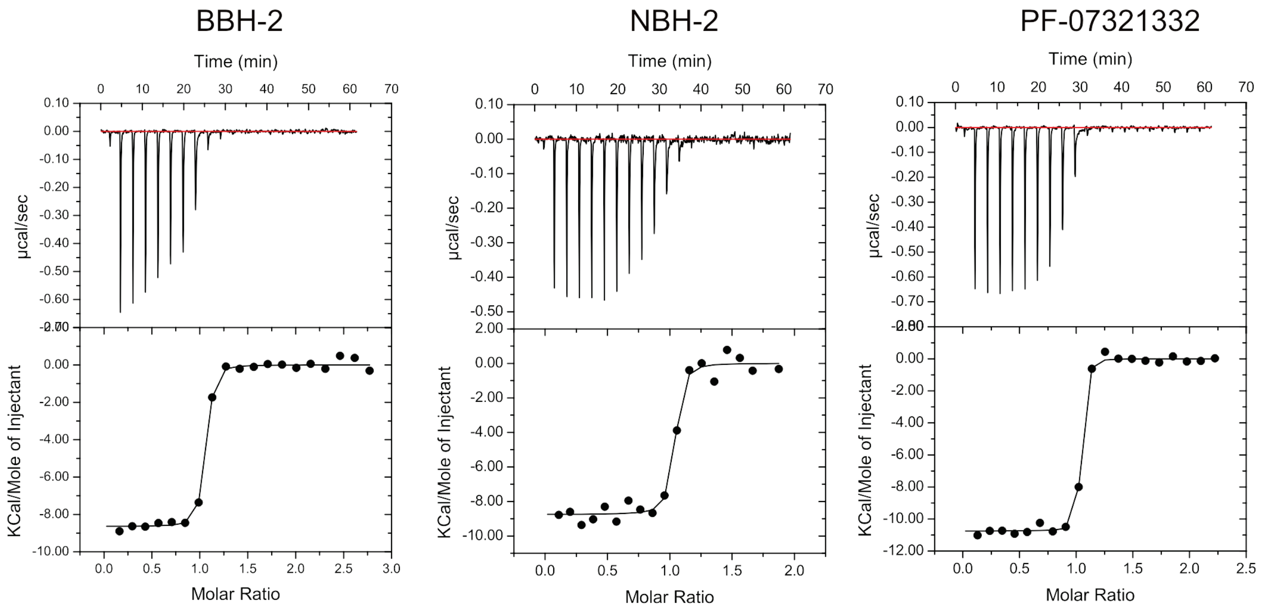

Supplementary Figure 4. Binding isotherms for the interaction of BBH-2, NBH-2 and PF-07321332 with M^{pro}.

Titrations were carried out in 25 mM Tris-HCl, pH 7.6, 20 mM NaCl, 1 mM TCEP and DMSO not exceeding 1.5% at 28°C. Thermodynamic parameters are listed in Table 2. Values listed for BBH-2 were derived from duplicate titrations (Table 1) and one of the plots is shown. Source data are provided as a Source Data file.

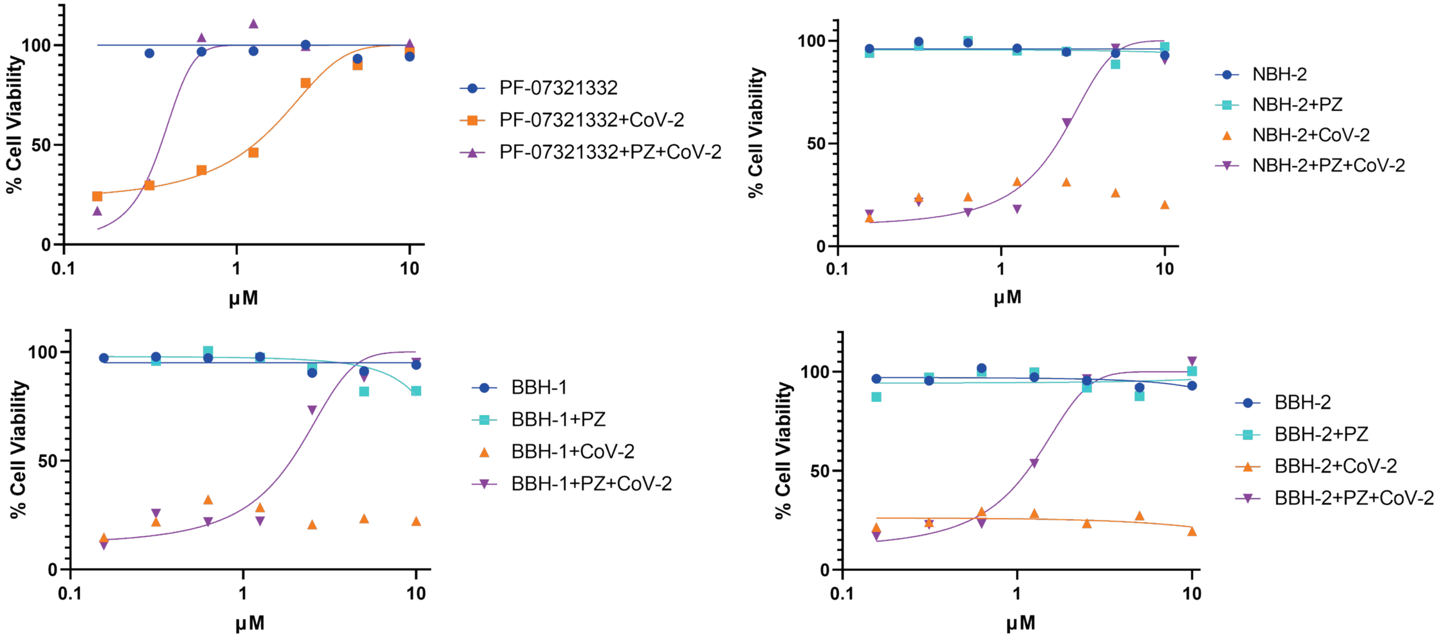

Supplementary Figure 5. Cytotoxicity and antiviral activity of the selected molecules against SARS-CoV-2. Seven concentrations of each molecule were tested in the presence or absence of SARS-CoV-2 in a cell-based assay in a 384-well plate with Vero E6 TMPRSS cells. Compounds were evaluated in a dose response format starting at 10 μM and 6 additional 2-fold dilutions in duplicate. The cytotoxicity of compounds was tested either alone or in the presence of the P-glycoprotein inhibitor CP-100356 at 2 μM. The antiviral activity was also assessed with the compound alone or in the presence of 2 μM CP-100356 and SARS-CoV-2. Following incubation for 48 hours at 5% CO_2 and 37ºC, the percent cell viability was measured with CellTiterGlo. Signals were read with an EnVision® 2105 multimode plate reader. Cells alone (positive control) and cells plus virus (negative control) were set to 100% and 0% cell viability to normalize the data from the compound testing. Data were normalized to cells (100%) and virus (0%) plus cells. Each concentration was tested in duplicate. PZ = CP-100356. Source data are provided as a Source Data file.

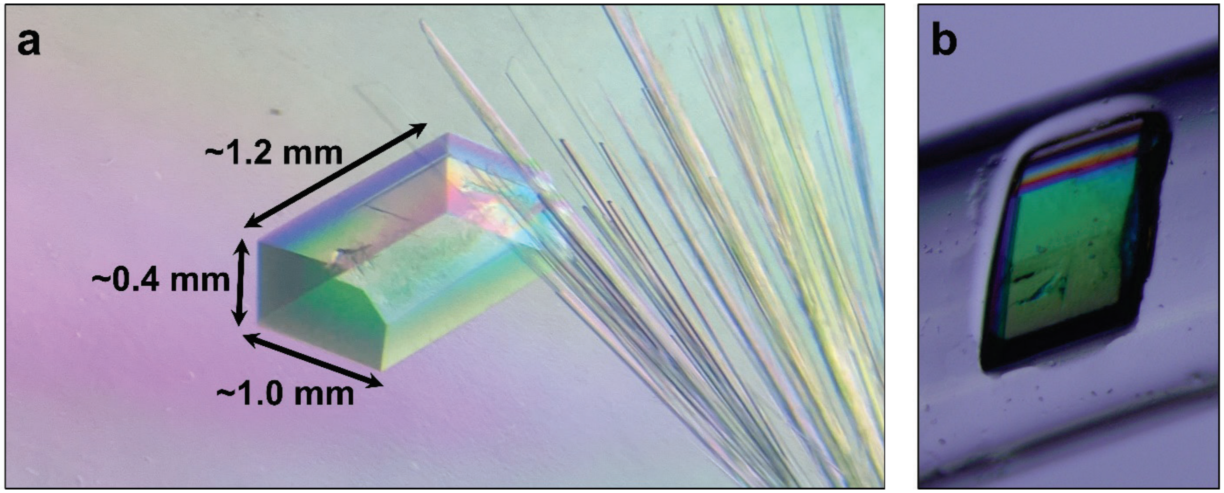

Supplementary Figure 6. Deuterated M^{pro}/BBH-1 crystal used for neutron diffraction

a) The M^{pro}/BBH-1 crystal used for neutron diffraction grew to ~0.5 mm^3 in a well containing needle-like crystal aggregates before b) being mounted in a fused quartz capillary.

Supporting Materials and Methods

Materials. All purchased reagents were at least 95% pure and were used as received from the suppliers without further purification unless otherwise noted. The narlaprevir fragment (1*R*,2*S*,5*S*)-3-((*S*)-2-(3-(1-((*tert*-butylsulfonyl) methyl)cyclohexyl)ureido)-3,3-dimethylbutanoyl)-6,6-dimethyl-3-azabicyclo[3.1.0]hexane-2-carboxylic acid was purchased from Synthonix (lot#5102, >98% purity).

Nuclear Magnetic Resonance (NMR). NMR spectra were obtained at the Center for Nanophase Materials Sciences on a Bruker Avance NEO NMR console coupled to a 11.74 T actively shielded magnet (Magnex Scientific/Varian) operating at 499.717 MHz for proton. All spectra were acquired at 298 K in either $CDCl_3$ (7.27 ppm ^{1}H reference and 77.23 ppm ^{13}C reference) or acetone-d_6 (2.05 ppm ^{1}H reference and 29.92 ppm ^{13}C reference). Assignments were confirmed using a combination of proton, COSY, carbon, carbon APT and HSQC experiments.

MALDI-ToF Mass Spectrometry (MALDI-ToF MS). Mass spectra were obtained at the Center for Nanophase Materials Sciences on a Bruker Autoflex Speed in positive ion reflectron mode using DCTB (trans-2-[3-(4-tert-Butylphenyl)-2-methyl-2-propenylidene]malononitrile) as the matrix. The matrix was prepared in THF at 60mg/mL and 0.5 μL spotted on the target surface and allowed to dry. 0.5 μL of the corresponding analyte solution (as given) was spotted on top of the crystallized DCTB and allowed to dry. Calculated and observed masses were compared to confirm the desired analyte.

Synthesis of Intermediates and Inhibitors

The boceprevir fragment (1*R*,2*S*,5*S*)-3-((*S*)-2-(3-(*tert*-butyl)ureido)-3,3-dimethylbutanoyl)-6,6-dimethyl-3-azabicyclo[3.1.0]hexane-2-carboxylic acid was synthesized following the procedures described in Bhalerao et al. 2015.[1] The benzothiazole intermediate *tert*-butyl ((*S*)-1-(benzo[*d*]thiazol-2-yl)-1-oxo-3-((*S*)-2-oxopyrrolidin-3-yl)propan-2-yl)carbamate was prepared as described by Thanigaimalai et al. 2013.[2]

***tert*-butyl ((*S*)-1-amino-1-oxo-3-((*S*)-2-oxopyrrolidin-3-yl)propan-2-yl)carbamate**

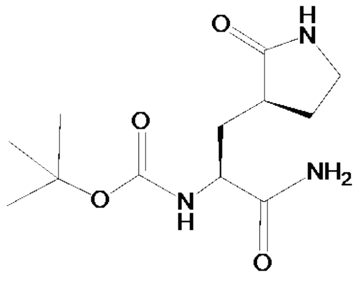

Methyl (*S*)-2-((*tert*-butoxycarbonyl)amino)-3-((*S*)-2-oxopyrrolidin-3-yl)propanoate (0.572 g, 2.0 mmol) was dissolved in 10 mL methanol, and 10 mL concentrated aqueous ammonia added dropwise with stirring at ambient temperature. After stirring overnight, the volatiles were removed by rotary evaporation. A few milliliters of benzene were added to the tacky solid residue, the solid triturated, and the benzene frozen. The frozen benzene was sublimed off under high vacuum to obtain the compound as a white powder in quantitative yield. NMR analysis revealed the product to be of sufficient purity to use in the next step without further purification. ^{1}H NMR (acetone-d_6): δ 7.20, (br s, 1H, –NH_2), 7.10 (br s, 1H, –NH_2), 6.63 (br s, 1H, lactam N*H*), 6.45 (br d, *J* = 5.3 Hz, 1H, N*H*CHC(O)NH_2), 4.22 (m, 1H, –C*H*C(O)NH_2), 3.38-3.24 (m, 2H, –$CH_aH_bCHCH_cH_dC\mathit{H}_2$NH–lactam), 2.50-2.31 (overlapping m, 2H, –$CH_aH_bC\mathit{H}C\mathit{H}_cH_dCH_2$NH–), 2.15-1.99 (m, 1H, –$C\mathit{H}_aH_bCHCH_cH_dCH_2$NH–), 1.87-1.71 (m, 2H, –$CH_a\mathit{H}_bCHCH_c\mathit{H}_dCH_2$NH–), 1.41 (s, 9H, ^{t}BuMe). ^{13}C{^{1}H} NMR (acetone-d_6): δ 180.2 (lactam *C*=O), 175.1 (–*C*(O)NH_2), (156.5 (–*C*(O)OC$(CH_3)_3$), 79.2 (–C(O)O*C*$(CH_3)_3$), 53.9 (–*C*HC(O)NH_2), 40.8 (–NH*C*H_2–lactam), 39.0 (–*C*H lactam), 35.2 (–*C*H_2CHC(O)NH_2), 29.0 (–NHCH_2*C*H_2– lactam), 28.7, (OC(*C*$H_3)_3$).

***tert*-butyl ((*S*)-1-cyano-2-((*S*)-2-oxopyrrolidin-3-yl)ethyl)carbamate**

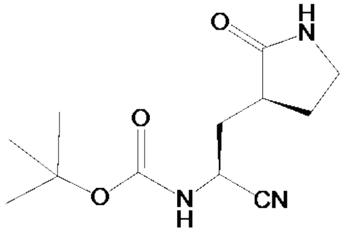

A modification of the procedure described by Moreno-Cinos et al. 2019[3] was followed. The above *tert*-butyl ((*S*)-1-amino-1-oxo-3-((*S*)-2-oxopyrrolidin-3-yl)propan-2-yl)carbamate (0.555 g, 2.0 mmol) was dissolved in 6.5 mL dry dichloromethane. To this solution with stirring at ambient temperature was added slowly dropwise a solution of 1-methoxy-*N*-(triethylammonio)sulfonylmethanimidate (Burgess Reagent, 0.958 g, 4.0 mmol) dissolved in 13.5 mL dry dichloromethane. The reaction flask was covered with a Teflon stopper and stirred at ambient temperature for 24 h. The reaction mixture was then washed with 1% acetic acid (2 x 20 mL), followed by brine (2 x 20 mL),[3] and dried through a column of anhydrous sodium sulfate. The solvent was removed to reveal a tacky solid. NMR showed that the product still contained a significant amount of the triethylammonium (methoxycarbonyl)sulfamate byproduct of the Burgess reagent. To better facilitate the removal of this salt, the crude product was then dissolved in 30 mL dichloromethane and the solution washed with 5% sodium bicarbonate solution (3 x 20 mL), followed by brine (20 mL). After drying as above, removal of the solvent afforded the product (0.295 g, 58%) at about 95% purity. ^{1}H NMR ($CDCl_3$): δ 6.63 (br s, 1H, lactam N*H*), 5.85 (br d, *J* = 7.7 Hz, 1H, N*H*CHCN), 4.70 (m, 1H, –C*H*CN), 3.42-3.35 (m, 2H, –$CH_aH_bCHCH_cH_dCH_2$NH–lactam), 2.55-2.42 (overlapping m, 2H, –$CH_aH_bCHCH_cH_dCH_2$NH–), 2.33-2.27 (m, 1H, –$CH_aH_bCHCH_cH_dCH_2$NH–), 1.98-1.84 (overlapping m, 2H, –$CH_aH_bCHCH_cH_dCH_2$NH–), 1.47 (s, 9H, ^{t}BuMe). ^{13}C{^{1}H} NMR ($CDCl_3$): δ 178.9 (lactam *C*=O), 154.9 (–*C*(O)OC$(CH_3)_3$), 119.2 (–*C*N), 81.4 (–C(O)O*C*$(CH_3)_3$), 41.3 (–*C*HCN), 40.6 (–NHCH_2– lactam), 38.0 (–*C*H lactam), 34.7 (–CH_2CHCN), 28.54 (–NHCH_2CH_2– lactam), 28.46, (OC$(CH_3)_3$).

(1*R*,2*S*,5*S*)-3-((*S*)-2-(3-(*tert*-butyl)ureido)-3,3-dimethylbutanoyl)-*N*-((*S*)-1-cyano-2-((*S*)-2-oxopyrrolidin-3-yl)ethyl)-6,6-dimethyl-3-azabicyclo[3.1.0]hexane-2-carboxamide (BBH-2)

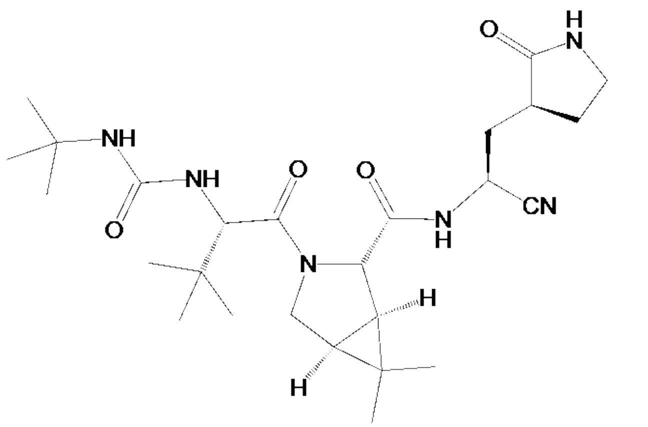

(*S*)-2-amino-3-((*S*)-2-oxopyrrolidin-3-yl)propanenitrile hydrochloride was prepared by treatment of a solution of *tert*-butyl ((*S*)-1-cyano-2-((*S*)-2-oxopyrrolidin-3-yl)ethyl)carbamate in dichloromethane with HCl in 1,4-dioxane (4 M) following the procedure described in Yang et al. 2006.[4] A sample of (*S*)-2-amino-3-((*S*)-2-oxopyrrolidin-3-yl)propanenitrile hydrochloride (29.0 mg, 0.15 mmol) was then dissolved in 5 mL dichloromethane along with (1*R*,2*S*,5*S*)-3-((*S*)-2-(3-(*tert*-butyl)ureido)-3,3-dimethylbutanoyl)-6,6-dimethyl-3-azabicyclo[3.1.0]hexane-2-carboxylic acid (37.5 mg, 0.10 mmol) and (2-(1*H*-benzotriazol-1-yl)-1,1,3,3-tetramethyluronium hexafluorophosphate (HBTU, 58.0, 0.15 mmol). To this solution was added *N*,*N*-diisopropylethylamine (DIPEA, 53.5 μL, 0.30 mmol) under nitrogen.[5,6] The resulting solution was stirred at room temperature overnight, concentrated and subjected to chromatography on silica gel (ethyl acetate, $R_f = 0.3$ in ethyl acetate). The fractions containing the desired product were combined, concentrated by rotary evaporation, and dried *in vacuo* to afford a white solid as the product (18.5 mg, 36%). ^{1}H NMR (acetone-d_6): δ 8.20 (br d, J = 7.6 Hz, 1H, N*H*CHCN), 6.81 (br s, 1H, lactam N*H*), 5.55 (br s, 1H, ^{t}BuN*H*C(O)), 5.43 (br d, J = 9.8 Hz, 1H, N*H*CH-^{t}Bu), 5.10-5.05 (m, 1H, C*H*-CN), 4.30 (d, J = 9.8 Hz, 1H, NHC*H*-^{t}Bu), 4.22 (s, 1H), 4.09 (d, J = 10.0 Hz, 1H), 3.93-3.90 (dd, J_1 = 5.3 Hz, J_2 = 10.0 Hz, 1H), 3.32-3.22 (overlapping m, 2H), 2.61-2.54 (m, 1H), 2.35-2.27 (overlapping m, 2H), 1.91-1.78 (overlapping m,

2H), 1.55-1.52 (m, 1H), 1.38 (d, J = 7.4 Hz, 1H), 1.24 (s, 9H, ^{t}BuMe), 1.04 (s, 3H, –CH_3), 0.96 (s, 9H, ^{t}BuMe), 0.90 (s, 3H, –CH_3). $^{13}C\{^1H\}$ NMR (acetone-d_6): δ 178.8 (lactam *C*=O,) 172.6, 172.2 (–CH(^{t}Bu)*C*(O)NCH*C*(O)NHCHCN–), 158.4 (–NH*C*(O)NH–), 120.1 (–*C*N), 61.3, 58.2, 50.4, 48.6, 40.6, 39.4, 38.1, 35.7, 35.2, 31.6, 29.6 (^{t}BuMe, underneath acetone-d_6 pentet, can be visualized using APT), 28.9, 28.7, 27.0 (^{t}BuMe), 26.5 (CH_3), 20.0, 13.2 (CH_3). MALDI-ToF (*m/z*): $C_{28}H_{42}N_6NaO_4^+$ $[M + Na]^+$ calc'd, 525.316; found, 525.237.

(1*R*,2*S*,5*S*)-3-((*S*)-2-(3-(1-((*tert*-butylsulfonyl)methyl)cyclohexyl)ureido)-3,3-dimethyl-butanoyl)-*N*-((*S*)-1-cyano-2-((*S*)-2-oxopyrrolidin-3-yl)ethyl)-6,6-dimethyl-3-azabicyclo [3.1.0]hexane-2-carboxamide (NBH-2)

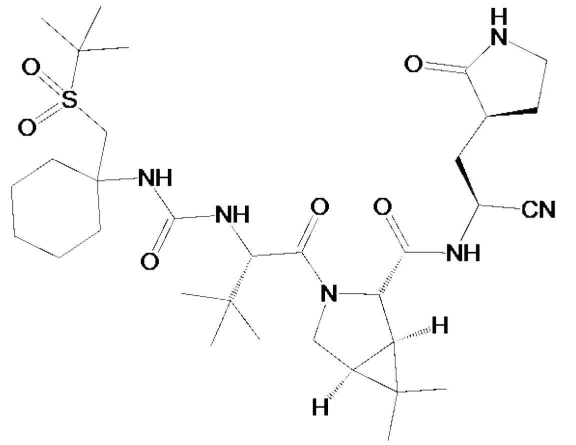

A procedure similar to that described by Yang et al 2006[4] was followed. A 10-mL round bottom flask was charged with *tert*-butyl ((*S*)-1-cyano-2-((*S*)-2-oxopyrrolidin-3-yl)ethyl)carbamate (27 mg, 0.10 mmol) and a small stir bar. To this was added 250 µL of 4 M HCl in 1,4-dioxane. After stirring at room temperature for 15 min, the slurry was diluted with an addition 1.0 mL of dry 1,4-dioxane, and an addition 200 µL of 4 M HCl in 1,4-dioxane added. After stirring for an additional 45 min, the volatiles were removed *in vacuo*, and 2 mL dry dichloromethane added, forming a suspension. The suspension was cooled in an ice bath, and 45 µL *N*-methylmorpholine (NMM) added with stirring. The solid begins to dissolve and an additional 22 µL NMM was added and stirring continued at 0 ºC for an additional 15 min, during which time all the solids

dissolved. This solution was then added to a solution of (1*R*,2*S*,5*S*)-3-((*S*)-2-(3-(1-((*tert*-butylsulfonyl)methyl)cyclohexyl)ureido)-3,3-dimethylbutanoyl)-6,6-dimethyl-3-azabicyclo[3.1.0]hexane-2-carboxylic acid (53 mg, 0.10 mmol), EDC•HCl (23 mg, 0.12 mmol), and HOBt•H_2O (16.8 mg, 0.11 mmol) in 1 mL dichloromethane, which had been previously prepared and stirred for 20 min. This reaction mixture was stirred at ambient temperature overnight. The solvent was then removed under vacuum, and the residue dissolved in ethyl acetate (20 mL). The solution was washed successively with 5% citric acid (2 x 10 mL), 5% sodium bicarbonate (2 x 10 mL), and brine (10 mL). After drying through a short column of granular anhydrous sodium sulfate, the solvent was removed under vacuum to afford the crude product (44 mg). This material was chromatographed using 95:5 ethyl acetate:methanol/SiO_2, which produced fractions containing 30.3 mg (46 %) that ranged in purity from 80 to 90%, on the basis of NMR and MALDI analyses. One ca. 90% pure fraction (4.5 mg) was used for crystallographic studies. Another ca. 90% pure fraction (9.8 mg) was then re-chromatographed on a pipet column using 2:1 cyclohexane:2-propanol/SiO_2, resulting in several fractions totaling 7.2 mg that were ≥93% pure, as analyzed by NMR. Material from these fractions were used for ITC and cell assay studies. ^{1}H NMR (acetone-d_6): δ 8.20 (br d, J = 7.4 Hz, 1H, N*H*CHCN), 6.80 (br s, 1H, lactam N*H*), 5.85 (br d, J = 9.3 Hz, 1H, N*H*CH-^{t}Bu), 5.55 (br s, 1H, CyN*H*C(O)), 5.11-5.05 (m, 1H, C*H*-CN), 4.35 (d, J = 9.3 Hz, 1H, NHC*H*-^{t}Bu), 4.23 (s, 1H), 4.03 (d, J = 10.1 Hz, 1H), 3.95-3.92 (dd, J_1 = 5.3 Hz, J_2 = 10.1 Hz, 1H), 3.86 (d, J = 13.6 Hz, 1H), 3.32-3.22 (overlapping m, 3H), 2.63-2.57 (m, 1H), 2.40-2.27 (overlapping m, 4H), 1.90-1.80 (m, 2H), 1.72-1.66 (m, 1H), 1.55-1.39 (overlapping m, 9H), 1.31 (s, 9H, ^{t}BuMe), 1.04 (s, 3H, –CH_3), 0.98 (s, 9H, ^{t}BuMe), 0.90 (s, 3H, –CH_3). ^{13}C{^{1}H} NMR (acetone-d_6): δ 178.8 (lactam *C*=O), 172.2 (overlapping –CH(^{t}Bu)*C*(O)NCH*C*(O)NHCHCN–), 158.1 (–NH*C*(O)NH–), 120.1 (–*C*N), 61.3, 60.3, 58.0, 55.0, 51.7, 48.6, 40.6, 39.4, 38.1, 36.1, 35.8, 35.65, 35.55, 31.6, 28.9, 28.8, 27.0 (^{t}BuMe), 26.5 (CH_3), 26.3, 23.4 (^{t}BuMe), 22.0 (2C in Cy), 20.0 (–*C*(CH_3)$_2$), 13.4 (CH_3). MALDI-ToF (*m/z*): $C_{33}H_{54}N_6NaO_6S^+$ [M + Na]$^+$ calc'd, 685.372; found, 685.343.

(1*R*,2*S*,5*S*)-N-((*S*)-1-(benzo[d]thiazol-2-yl)-1-oxo-3-((*S*)-2-oxopyrrolidin-3-yl)propan-2-yl)-3-((*S*)-2-(3-(tert-butyl)ureido)-3,3-dimethylbutanoyl)-6,6-dimethyl-3-azabicyclo[3.1.0]-hexane-2-carboxamide (BBH-1)

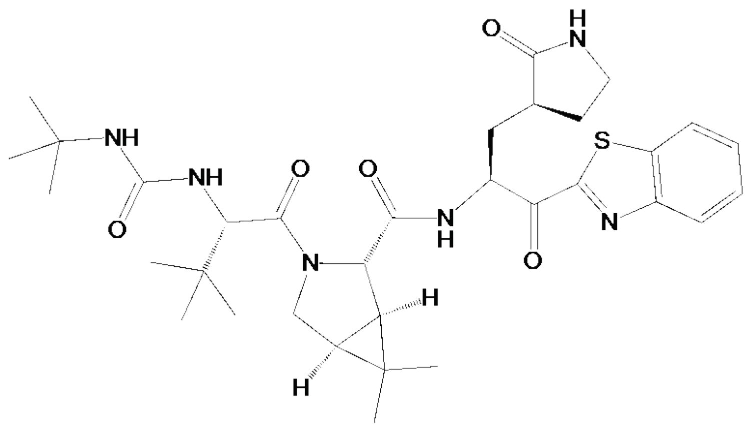

(*S*)-3-((*S*)-2-amino-3-(benzo[*d*]thiazol-2-yl)-3-oxopropyl)pyrrolidin-2-one hydrochloride was prepared by treatment of a solution of *tert*-butyl ((*S*)-1-(benzo[*d*]thiazol-2-yl)-1-oxo-3-((*S*)-2-oxopyrrolidin-3-yl)propan-2-yl)carbamate in dichloromethane with HCl in 1,4-dioxane (4 M) following the procedure described in Yang et al. 2006.[4] A sample of (*S*)-2-amino-3-((*S*)-2-oxopyrrolidin-3-yl)propanenitrile hydrochloride (133 mg, 0.41 mmol) was then dissolved in 5 mL dry dichloromethane along with (1*R*,2*S*,5*S*)-3-((*S*)-2-(3-(*tert*-butyl)ureido)-3,3-dimethylbutanoyl)-6,6-dimethyl-3-azabicyclo[3.1.0]hexane-2-carboxylic acid (150 mg, 0.41 mmol) and EDC•HCl (78 mg, 0.41 mmol), To this solution was added *N*,*N*-diisopropylethylamine (DIPEA, 172 µL, 0.96 mmol) under nitrogen. The resulting solution was stirred at room temperature overnight, concentrated and subjected to chromatography on silica gel (ethyl acetate, $R_f = 0.3$ in ethyl acetate). The fractions containing the desired product were combined, concentrated by rotary evaporation, and dried *in vacuo* to give a pale yellow solid as final product (yield 83 mg, 32%). This material was chromatographed using ethyl acetate/SiO_2, which produced fractions containing mixtures of rotamers[7] of varying ratios (the barrier to rotation is sufficiently high that some separation of the rotamers is possible, yielding fractions with differing ratios, though at equilibrium, prior to chromatography, the ratio appears to be about 3:1.) NMR analysis indicated fractions were generally ≥93

% pure, and separate peaks for certain resonances of the two rotamers can be observed in the carbon NMR at ambient temperature.[7] ^{1}H NMR (acetone-d_6): δ for both rotamers 8.28 (br d) overlaps with 8.27-8.22 (m, aromatic), 8.00 (br d), 7.72-7.65 (m, aromatic), 6.82-6.76 (overlapping br d), 5.87-5.81 (m), 5.56 (br d), 5.43 (br overlapping d), 4.48-4.42 (m), 4.38 (br s), 4.32 (br d), 4.28 (br s), 4.07 (br d), 3.97-3.88 (two pairs of dd), 3.36-3.21 (overlapping m), 2.78-2.70 (m), 2.58-2.55 (m), 2.54-2.48 (m), 2.37-2.30 (m), 2.21-2.11 (m), 2.02-1.91 (m), 1.80-1.73 (m), 1.54-1.27 (overlapping m), 1.26-1.25 (overlapping s from both rotamers, 9H, ^{t}BuMe), 1.03, 1.02 (overlapping s both rotamers, 3H, –CH_3), 0.97, 0.96 (overlapping s both rotamers,, 9H, ^{t}BuMe), 0.91, 0.90 (overlapping s both rotamers, 3H, –CH_3). ^{13}C{^{1}H} NMR (acetone-d_6): δ 193.91, 193.63 (benzothiazole carbonyl); 179.94, 179.53 (lactam *C*=O); 172.56, 172.51, 172.29, 172.13 (–CH(^{t}Bu)*C*(O)NCH*C*(O)NHCH–), 166.00, 165.63 (benzothiazole –S*C*=N–); 158.39, 158.34 (–NH*C*(O)NH–); 154.52 (benzothiazole Ar, both rotamers); 137.99, 137.96 (benzothiazole Ar); 129.07, 128.99 (benzothiazole Ar); 128.30, 128.26 (benzothiazole Ar); 126.38, 126.34 (benzothiazole Ar); 123.80, 123.76 (benzothiazole Ar); 61.63, 61.22; 58.22, 58.18 (–*C*H-^{t}Bu); 55.39, 54.88; 50.35, 50.33 (NH-*C*(Me)$_3$); 48.60, 48.53; 40.76 (both rotamers); 39.21, 38.94 (–*C*H lactam); 39.06 (–CH*C*(Me)$_3$, both rotamers); 35.28; 34.5, 32.99; 31.96, 31.85; 29.68 (^{t}BuMe, underneath acetone-d_6 pentet, can be visualized using APT, both rotamers), 28.79, 28.76; 27.05 (^{t}BuMe both rotamers); 26.65, 26.60 (CH_3); 26.40, 23.02; 19.86, 19.83 (–*C*(CH_3)$_2$); 13.26, 13.23 (CH_3). MALDI-ToF (*m/z*): $C_{33}H_{46}N_6NaO_5S^+$ $[M + Na]^+$ calc'd, 661.314; found, 661.285

References:

1. Bhalerao, D. S.; Arkala, A. K. R.; Madhavi, Y. V.; Nagaraju, M.; Gade, S. R.; Kumar, U. K. S.; Bandichor, R.; Dahanukar, V. H. "Synthesis and Process Optimization of Boceprevir: A Protease Inhibitor Drug." *Org. Process. Res. Dev.* **2015**, *19*, 1559-1567.

2. Thanigaimalai, P.; Konno, S.; Yamamoto, T.; Koiwai, Y.; Taguchi, A.; Takayama, K.; Yakushiji, F.; Akaji, K.; Chen, S.-E.; Naser-Tavakolian, A.; Schön, A.; Freire, E.; Hayashi, Y. "Development of potent dipeptide-type SARS-CoV 3CL protease inhibitors with novel P3 scaffolds: Design, synthesis, biological evaluation, and docking studies." *Eur. J. Med. Chem.* **2013**, *68*, 372-384.

3. Moreno-Cinos, C.; Sasetti, E.; Salado, I. G.; Witt, G.; Benramdane, S.; Reinhardt, L.; Cruz, C. D.; Joossens, J.; Van der Veken, P.; Brötz-Oesterhelt, H.; Tammela, P.; Winterhalter, M.; Gribbon, P.; Windshügel, B.; Augustyns, K. "α-Amino Diphenyl Phosphonates as Novel Inhibitors of *Escherichia coli* ClpP Protease." *J. Med. Chem.* **2019**, *62*, 774-797.

4. Yang, S.; Chen, S.-J.; Hsu, M.F.; Wu, J.-D.; Tseng, C.-T. K.; Liu, Y.-F.; Chen, H.-C.; Kuo, C.-W.; Wu, C.-S.; Chang, L.-W.; Chen, W.-C.; Liao, S.-Y.; Change, T.-Y., Hung, H.-H.; Shr, H.-L.; Liu, C.-Y.; Huang, Y.-A.; Chang, L.-Y.; Hsu, J.-C.; Peters, C. J.; Wang, A. H.-J.; Hsu, M.-C. "Synthesis, Crystal Structure, Structure-Activity Relationships, and Antiviral Activity of a Potent SARS Coronavirus 3CL Protease Inhibitor." *J. Med. Chem.* **2006**, *49*, 4971-4980.

5. Dai, W.; Zhang, B.; Jiang, X.-M.; Su, H.; Li, J.; Zhao, Y.; Xie, X.; Jin, Z.; Peng, J.; Liu, F.; Li, C.; Li, Y.; Bai, F.; Wang, H.; Cheng, X.; Cen, X.; Hu, S.; Yang, X.; Wang, J.; Liu, X.; Xiao, G.; Jiang, H.; Rao, Z.; Zhang, L.-K.; Xu, Y.; Yang, H.; Liu, H. "Structure-based design of antiviral drug candidates targeting the SARS-CoV-2 main protease." *Science* **2020**, *368*, 1331-1335.

6. Zhang, L.; Lin, D.; Kusov, Y.; Nian, Y.; Ma, Q.; Wang, J.; von Brunn, A.; Leyssen, P.; Lanko, K.; Neyts, J.; de Wilde, A.; Snijder, E. J.; Liu, H.; Hilgenfeld, R. "α-Ketoamides as Broad-Spectrum Inhibitors of Coronavirus and Entervirus Replication: Structure-Based Design, Synthesis, and Activity Assessment." *J. Med. Chem.* **2020**, *63*, 4562-4578.

7. Owen, D. R.;Allerton, C. M. N.; Anderson, A. S.; Aschenbrenner, L.; Avery, M.; Berritt, S.; Boras, B.; Cardin, R. D.; Carlo, A.; Coffman, K. J.; Dantonio, A.; Di, L.; Eng, H.; Fere, R.; Gajiwala, K. T.; Gibson, S. A.; Greasley, S. E.; Hurst, B. L.; Kadar, E. P.; Kalgutkar, A. S.; Lee, J. C.; Lee, J.; Liu, W.; Mason, S. W.; Noell, S.; Novak, J. J.; Obach, R. S.; Ogilvie, K.; Patel, N. C.; Pettersson, M.; Rai, D. K.; Reese, M. R.; Sammons, M. F.; Sathish, J. G.; Singh, R. S. P.; Steppan, C. M.; Stewart, A. E.; Tuttle, J. B.; Updyke, L.; Verhoest, P. R.; Wei, L.; Yang, Q.; Zhu, Y. *Science*, **2021**, *374*, 1586-1593.

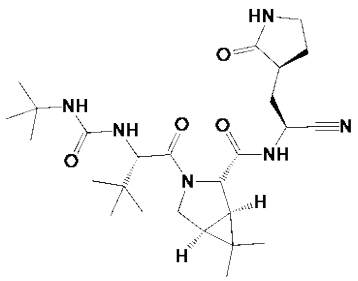

BBH-2

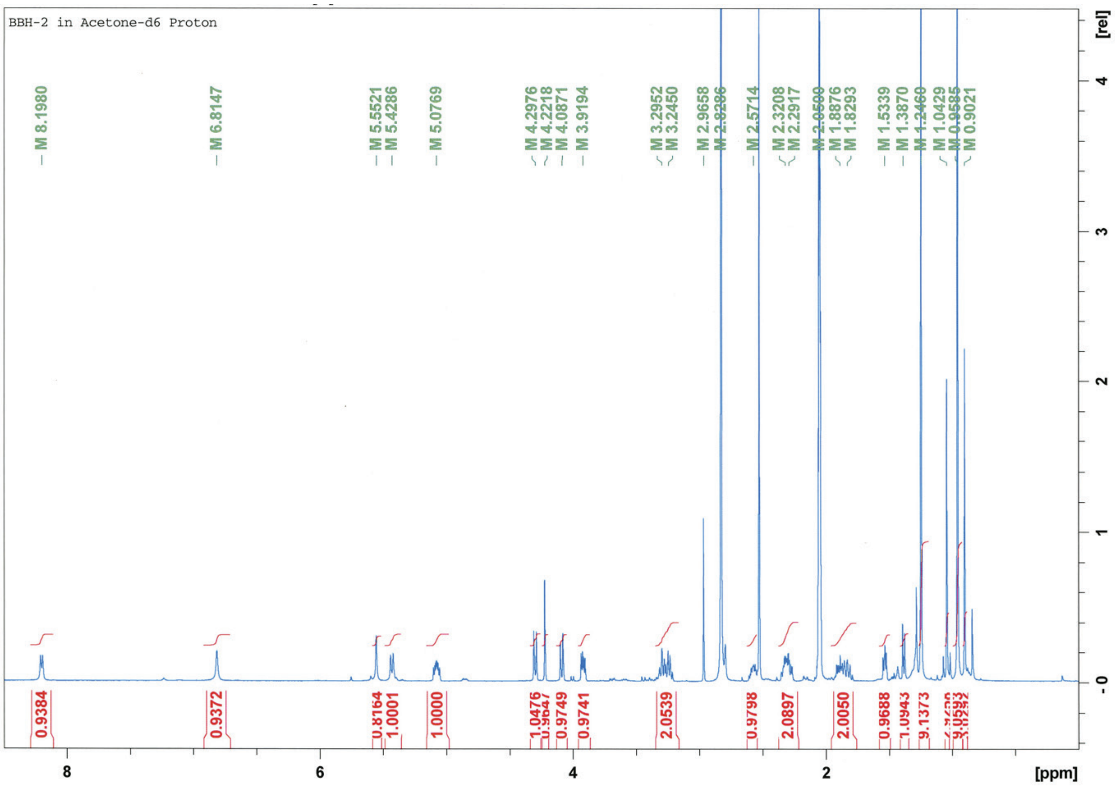
BBH-2 in Acetone-d6 Proton
M 8.1980
M 6.8147
M 5.5521
M 5.4286
M 5.0769
M 4.2976
M 4.2218
M 4.0871
M 3.9194
M 3.2952
M 3.2450
M 2.9658
M 2.5714
M 2.3208
M 2.2917
M 1.8876
M 1.8293
M 1.5339
M 1.3870
M 1.0429
M 0.9021
0.9384
0.9372
1.0001
1.0000
1.0476
0.9749
0.9741
2.0539
0.9798
2.0897
2.0050
0.9688
9.1373
[rel]
[ppm]

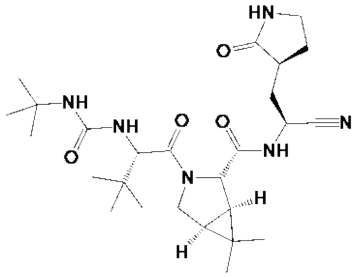

BBH-2

BBH-2 in Acetone-d6

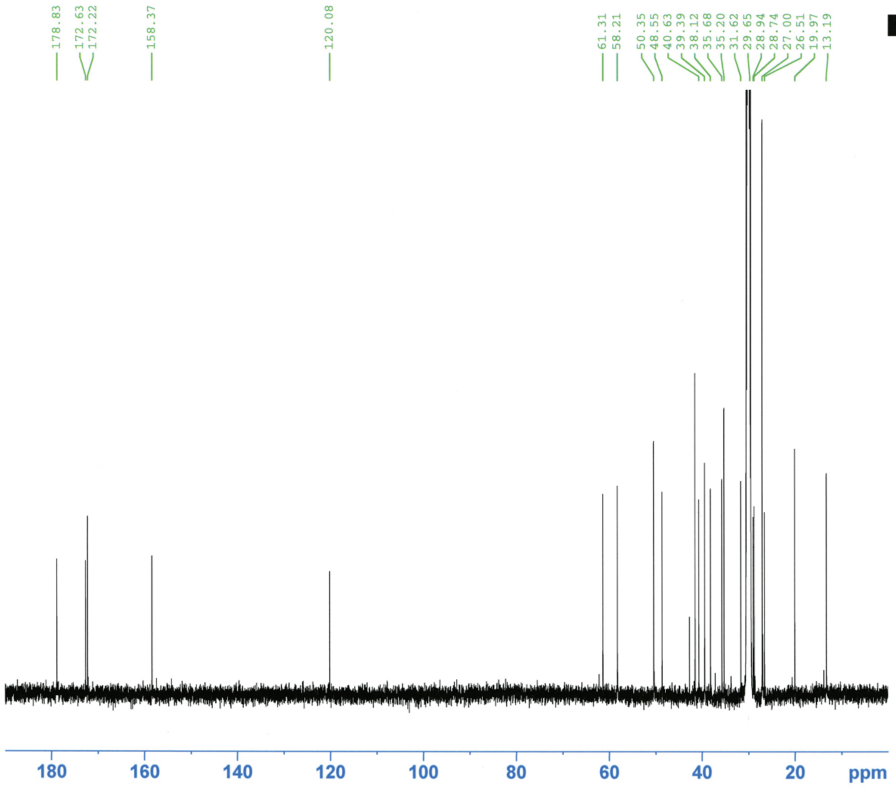

Current Data Parameters
NAME HL-3-102 BBH-CN
EXPNO 13
PROCNO 1

F2 - Acquisition Parameters

Date_	20210723	
Time	7.34	h
INSTRUM	Avance	
PROBHD	Z167419_0028 (	
PULPROG	zgpg30	
TD	65536	
SOLVENT	Acetone	
NS	16384	
DS	4	
SWH	30120.482	Hz
FIDRES	0.919204	Hz
AQ	1.0878977	sec
RG	101	
DW	16.600	usec
DE	6.50	usec
TE	298.0	K
D1	3.00000000	sec
D11	0.03000000	sec
TD0	16	
SFO1	125.6660020	MHz
NUC1	13C	
P0	3.00	usec
P1	9.00	usec
PLW1	104.00000000	W
SFO2	499.7169989	MHz
NUC2	1H	
CPDPRG[2	waltz65	
PCPD2	80.00	usec
PLW2	26.00000000	W
PLW12	0.22852001	W
PLW13	0.11494000	W

F2 - Processing parameters

SI	32768	
SF	125.6533133	MHz
WDW	EM	
SSB	0	
LB	1.00	Hz
GB	0	
PC	1.40	

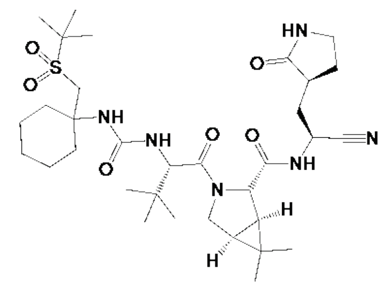

NBH-2

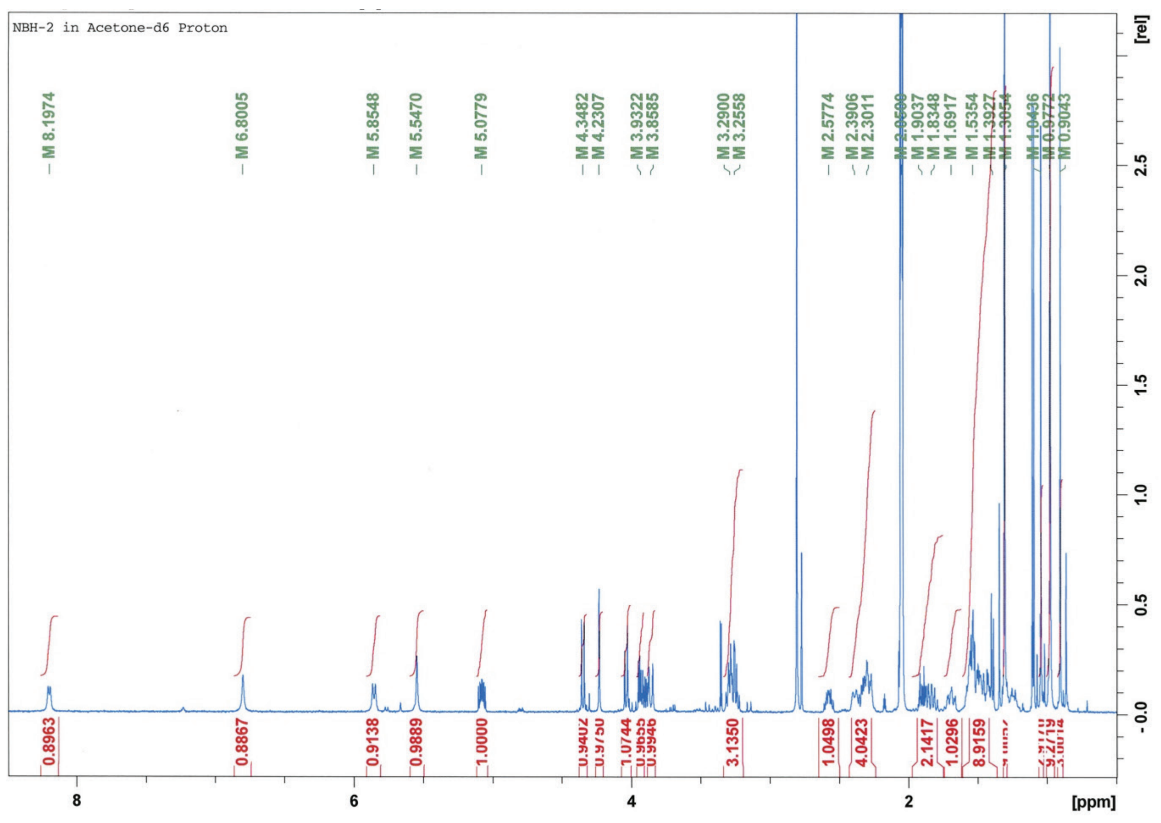
NBH-2 in Acetone-d6 Proton
M 8.1974
M 6.8005
M 5.8548
M 5.5470
M 5.0779
M 4.3482
M 4.2307
M 3.9322
M 3.8585
M 3.2900
M 3.2558
M 2.5774
M 2.3906
M 2.3011
M 1.9037
M 1.8348
M 1.6917
M 1.5354
M 0.9043
0.8963
0.8867
0.9138
0.9889
1.0000
1.0744
3.1350
1.0498
4.0423
2.1417
1.0296
8.9159
[rel]
[ppm]

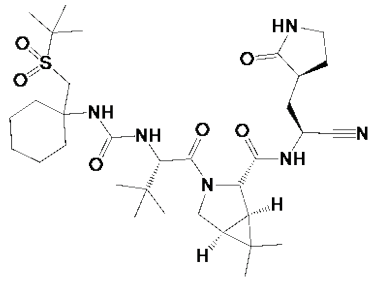

NBH-2

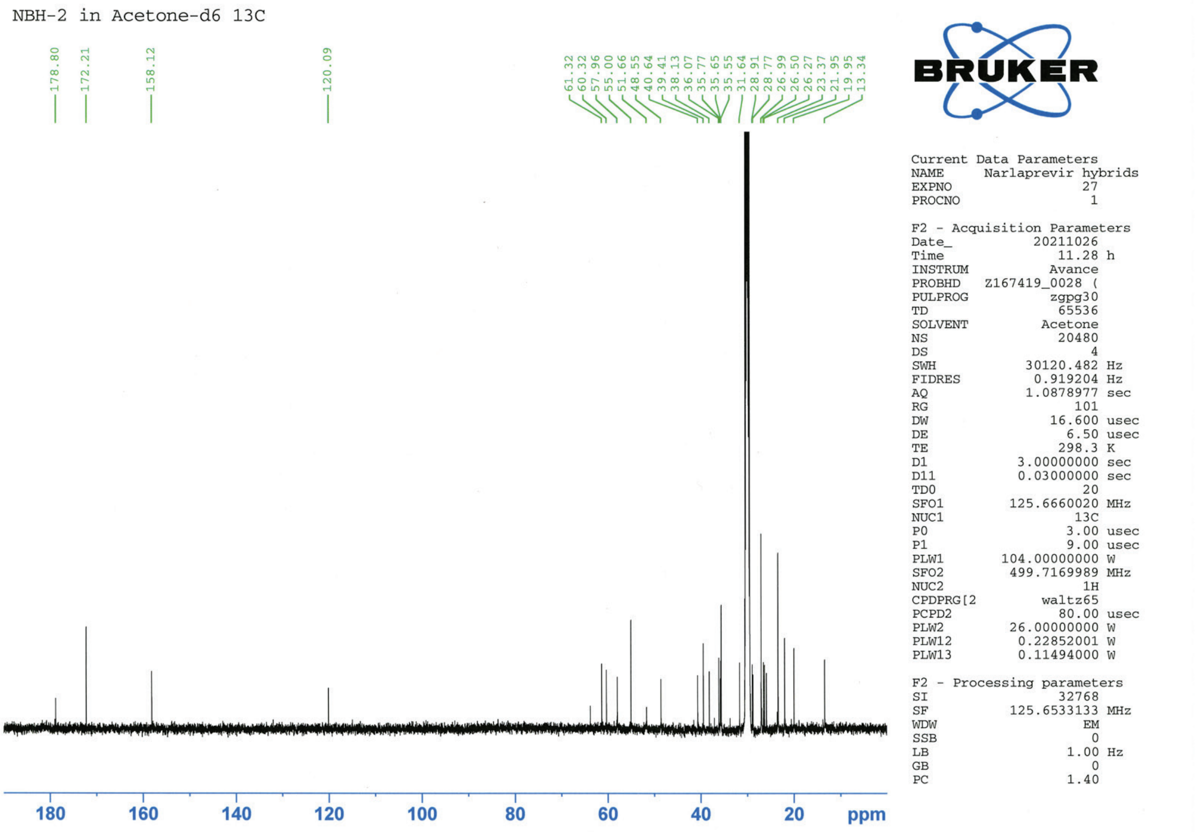
NBH-2 in Acetone-d6 13C
178.80
172.21
158.12
120.09
61.32
60.32
57.96
55.00
51.66
48.55
40.64
39.41
38.13
36.07
35.77
35.65
35.55
31.64
28.91
28.77
26.99
26.50
26.27
23.37
21.95
19.95
13.34
BRUKER
Current Data Parameters
NAME Narlaprevir hybrids
EXPNO 27
PROCNO 1
F2 - Acquisition Parameters
Date_ 20211026
Time 11.28 h
INSTRUM Avance
PROBHD Z167419_0028 (
PULPROG zgpg30
TD 65536
SOLVENT Acetone
NS 20480
DS 4
SWH 30120.482 Hz
FIDRES 0.919204 Hz
AQ 1.0878977 sec
RG 101
DW 16.600 usec
DE 6.50 usec
TE 298.3 K
D1 3.00000000 sec
D11 0.03000000 sec
TD0 20
SFO1 125.6660020 MHz
NUC1 13C
P0 3.00 usec
P1 9.00 usec
PLW1 104.00000000 W
SFO2 499.7169989 MHz
NUC2 1H
CPDPRG[2 waltz65
PCPD2 80.00 usec
PLW2 26.00000000 W
PLW12 0.22852001 W
PLW13 0.11494000 W
F2 - Processing parameters
SI 32768
SF 125.6533133 MHz
WDW EM
SSB 0
LB 1.00 Hz
GB 0
PC 1.40
180
160
140
120
100
80
60
40
20
ppm

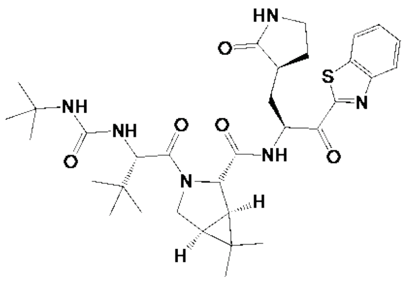

BBH-1

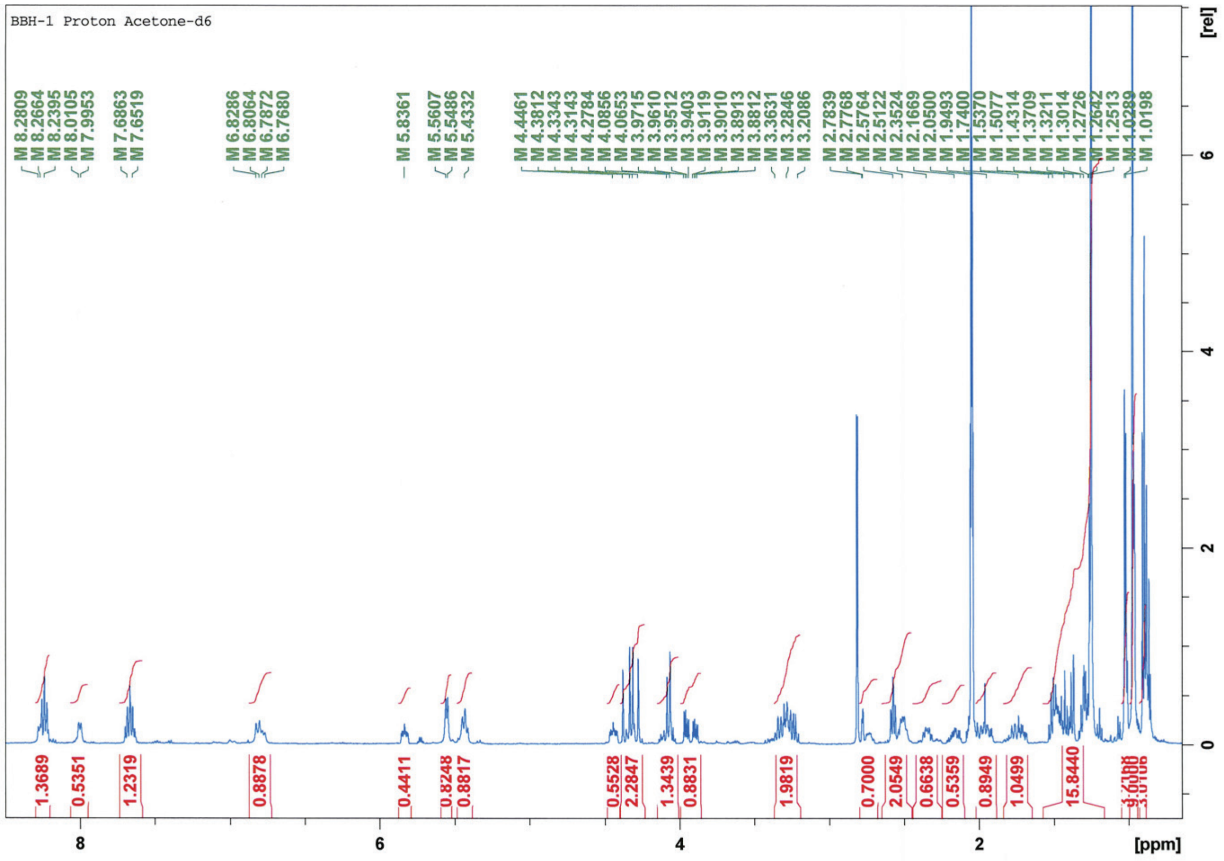

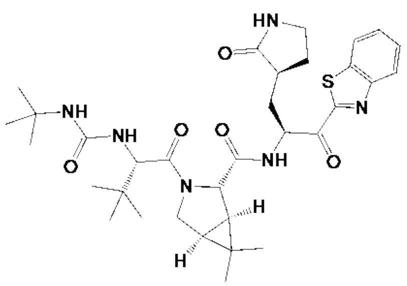

BBH-1

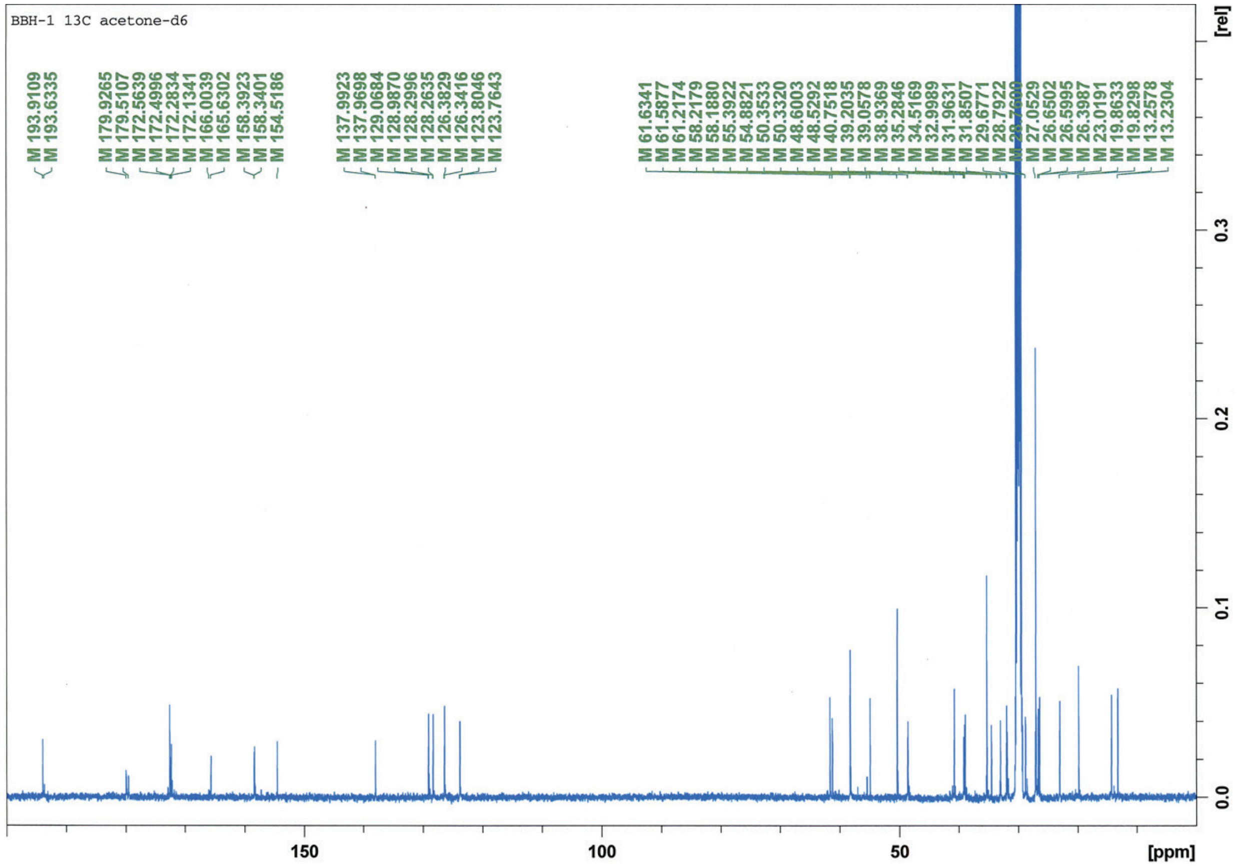

BBH-1 13C acetone-d6
M 193.9109
M 193.6335
M 179.9265
M 179.5107
M 172.5639
M 172.4996
M 172.2834
M 172.1341
M 166.0039
M 165.6302
M 158.3923
M 158.3401
M 154.5186
M 137.9923
M 137.9698
M 129.0684
M 128.9870
M 128.2996
M 128.2635
M 126.3829
M 126.3416
M 123.8046
M 123.7643
M 61.6341
M 61.5877
M 61.2174
M 58.2179
M 58.1880
M 55.3922
M 54.8821
M 50.3533
M 50.3320
M 48.6003
M 48.5292
M 40.7518
M 39.2035
M 39.0578
M 38.9369
M 35.2846
M 34.5169
M 32.9989
M 31.9631
M 31.8507
M 29.6771
M 28.7922
M 27.0529
M 26.6502
M 26.5995
M 26.3987
M 23.0191
M 19.8633
M 19.8298
M 13.2578
M 13.2304
[rel]
0.0
0.1
0.2
0.3
150
100
50
[ppm]

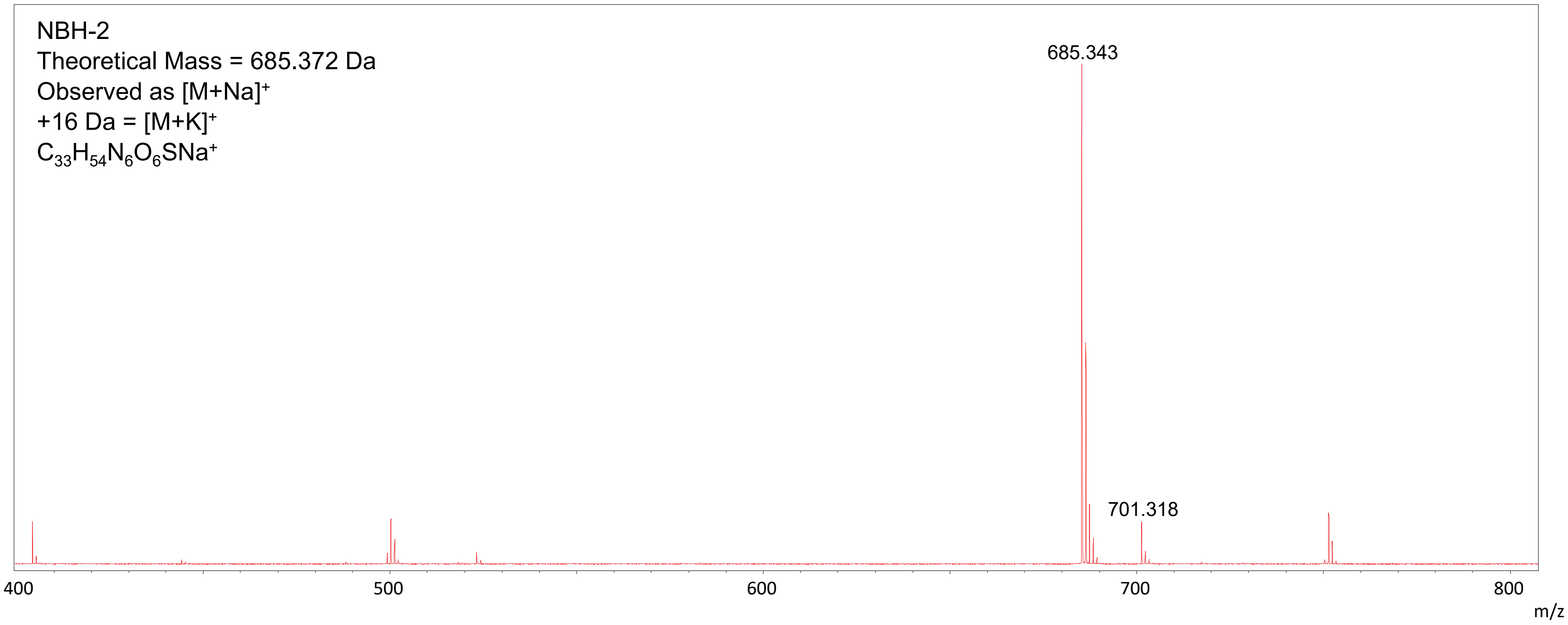

NBH-2
Theoretical Mass = 685.372 Da
Observed as [M+Na]+
+16 Da = [M+K]+
$C_{33}H_{54}N_6O_6SNa^+$
685.343
701.318
400
500
600
700
800
m/z

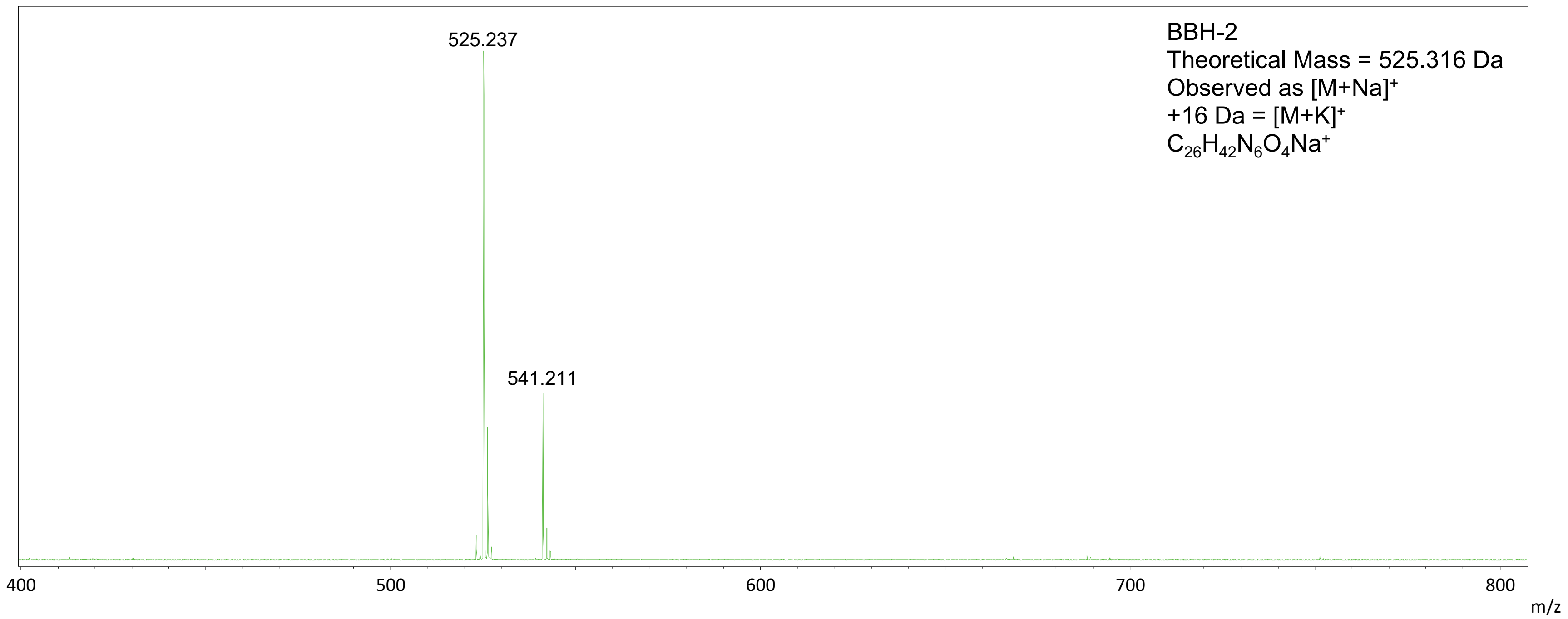
BBH-2
Theoretical Mass = 525.316 Da
Observed as [M+Na]$^+$
+16 Da = [M+K]$^+$
$C_{26}H_{42}N_6O_4Na^+$
525.237
541.211
400
500
600
700
800
m/z

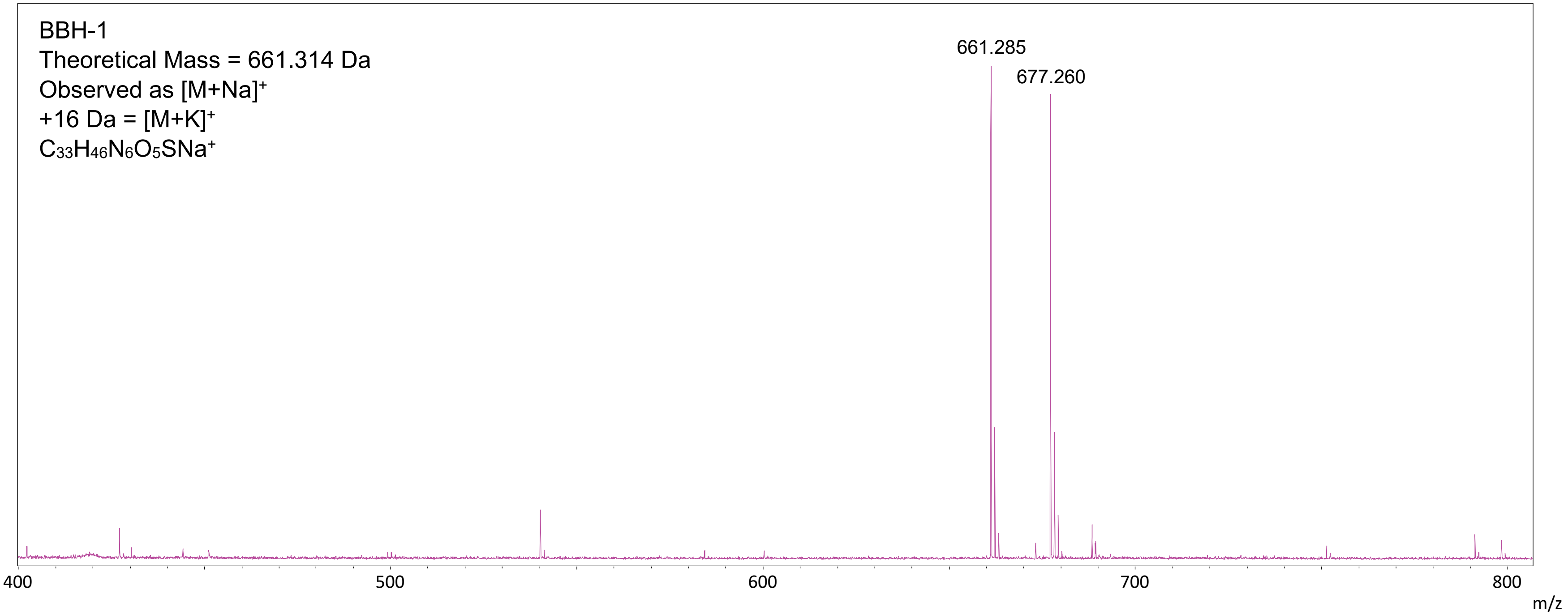
BBH-1
Theoretical Mass = 661.314 Da
Observed as [M+Na]+
+16 Da = [M+K]+
C33H46N6O5SNa+
661.285
677.260
400
500
600
700
800
m/z
